# A comparison of self-reported chronic disease, health awareness and behaviours in social housing residents: cross-sectional study of communities in Ontario and Quebec

**DOI:** 10.1186/s13104-024-06849-x

**Published:** 2024-07-30

**Authors:** Gina Agarwal, Melissa Pirrie, Christie Koester, Drashti Pete, Julia Antolovich, Ricardo Angeles, Francine Marzanek, Magali Girard, Janusz Kaczorowski

**Affiliations:** 1 Department of Family Medicine, McMaster University, David Braley Health Sciences Centre, 5th Floor, 100 Main Street West, Hamilton, ON L8P 1H6 Canada; 2 Department of Health Research Methods, Evidence, and Impact, McMaster University, Hamilton, ON Canada; 3 Le Centre de Recherche du Centre Hospitalier de l’Université de Montréal, Montréal, QC Canada; 4 Département de médecine de famille et médecine d’urgence, Université de Montréal, Montréal, QC Canada

**Keywords:** Chronic disease, Health behaviour, Quality of life, Social housing, Risk factors, Diabetes

## Abstract

**Objective:**

Social housing programs are integral to making housing more affordable to Canadian seniors living in poverty. Although the programs are similar across Canada, there may be inter-provincial differences among the health of residents that could guide the development of interventions. This study explores the health of low-income seniors living in social housing in Quebec and compares it with previously reported data from Ontario.

**Results:**

80 responses were obtained in Quebec to compare with the previously reported Ontario data (n = 599) for a total of 679 responses. More Ontario residents had access to a family doctor (p < 0.001). Quebec residents experienced less problems with self-care (p = 0.017) and less mobility issues (p = 0.052). The visual analog scale for overall health state was similar in both provinces (mean = 67.36 in Ontario and 69.23 in Quebec). Residents in Quebec smoked more cigarettes per day (p = 0.009). More residents in Ontario participated in moderate physical activity (p = 0.09), however, they also spent more time per day on the computer (p = 0.006).

## Introduction

In 2018, there were 216,000 Canadian seniors living in poverty [1]. Low-income seniors face poorer health outcomes than the general population, are less likely to be physically active, less likely to participate in health-promoting behaviours and more likely to engage in unhealthy behaviours [2].

Social housing programs are an integral part of government initiatives to make housing more affordable for those facing poverty and social inequities [3, 4]. Literature [5, 6] on low-income seniors in social housing in Canada highlights a higher prevalence of chronic disease and health risk factors, poorer mental health, lower health literacy, and poorer access to healthcare compared to the general population. Understanding the health challenges that low-income seniors living in social housing face is important so that interventions can be tailored and developed to address the needs of this increasingly vulnerable population.

Providing targeted services for this vulnerable and overlooked population is important, especially for pre-senior individuals (55 to 64 years old) who are often not eligible for pensions or benefits that are provided for seniors [7].

Social housing programs are similar across Canada in that they aim to help low-income older adults afford housing. However, there may be inter-provincial differences among the health of residents not captured in national data that could guide the development of interventions and support services to help improve their quality of life. According to the Canadian Community Health Survey (CCHS) comparing Quebec and Ontario, there are some differences and similarities in the prevalence of chronic disease, health risk factors and health-related quality of life between the provinces [8]. One potential reason for some of the differences may be due to the differing provincial standards for allowing priority access to social housing units. In Ontario, each region provides varying priority access for those in urgent situations, such as victims of domestic violence, homelessness, victims of human trafficking and terminal illness [9]. In contrast, Quebec employs the same priorities across the entire province, which includes victims of domestic violence, those who are mobility impaired [10], and people whose homes have been accidentally destroyed [11]. Therefore, interventions that are effective in improving the health of low-income seniors in Ontario may need to be adapted to the Quebec population.

From previous data collected in Ontario, we know that seniors in social housing have low health literacy [12], poor quality of life [13], and high rates of chronic disease [13]. This study explores the health of low-income seniors living in social housing in Quebec communities and compares it with what was previously reported in Ontario communities [14]. These findings will inform the adaptations of an existing Ontario health intervention for the Quebec context.

## Methods

### Study design

A cross-sectional study design was used to evaluate the health status, health behaviours, and health risk knowledge of older adults in social housing apartment buildings in Quebec. Data was collected using surveys that were completed in-person with older adults residing in four social housing buildings between March and April 2018 in two communities in Quebec. We calculated the sample size for a cross-sectional study using health literacy as the outcome. With a total number of 147 residents in all 4 buildings in Quebec and assuming 85–90% of residents will have poor health literacy, we needed a minimum of 72 residents (β = 0.8, α = 0.05).

This data was then compared to previously collected data from 30 social housing buildings in five communities in Ontario between January 2015 and November 2016.

### Participants

The survey was conducted in four social housing buildings located in Saint-Hubert and Longueuil, which are suburban areas of Montreal, Quebec. Participants were eligible for the study if they were current residents of these social housing buildings and at least 55 years old. The survey was advertised in common areas of the building with a ten-dollar gift card given as an incentive. In addition to a standard participant information letter outlining the study, details of the study were explained verbally to the participants before participants provided informed written consent and the survey was administered. Ethics approval was obtained by local Research Ethics Boards.

### Measures

The Health Awareness and Behaviour Tool (HABiT) used had been previously piloted with a sample of older adults in Ontario [15]. It was specifically designed for older adult populations in order to assess their health status [15]. It consisted of questions from other validated surveys such as the CCHS [8], Canadian Diabetes Risk Questionnaire (CANRISK) [16], the Canadian Hypertension Education Program (CHEP) [17], the EuroQual Five Dimensions using 3-Levels (EQ5D-3L) [18], and the Newest Vital Sign UK (NVS-UK) [19]. All measures were self-reported by the participants. The survey looks at a participant’s health knowledge, health-related quality of life, chronic disease (history and monitoring), health behaviours and health literacy. All study data was collected using paper surveys through face-to-face interviews in either French or English by trained research staff, paramedics and volunteers.

#### Health knowledge

There were eight diabetes knowledge statements and ten cardiovascular knowledge statements where the participant was asked to indicate if they felt the statement was true or false, using a 5-point Likert type scale (Definitely True, Maybe True, Not Sure or Don’t Know, Maybe False, and Definitely False). In the analysis, these responses were dichotomized into those who gave the correct response (either ‘definitely’ or ‘maybe’) and those who did not give a correct response (‘not sure or don’t know,’ the ‘maybe’ version of the wrong response, or the ‘definitely’ version of the wrong response.)

#### Health-related quality of life (HRQoL)

Participants were asked about their HRQoL using the EQ5D-3L. The EQ5D-3L includes questions about their mobility, self-care, usual activities, pain/discomfort and anxiety/depression. Self-reported health status was scored on a scale of 1 to 100.

#### Chronic disease history and monitoring

Questions were asked about the presence of any chronic diseases such as hypertension, diabetes, stroke, heart problems and cholesterol. Participants were also asked about the frequency of their blood pressure, cholesterol and blood sugar level measurement. Self-reported systolic and diastolic blood pressures were categorized as ‘high’ if ≥ 140 mmHg and ≥ 90 mmHg, respectively, according to the Hypertension Canada guidelines [17].

#### Diabetes risk score

In addition, the HABiT contains all questions from the CANRISK questionnaire, which calculates an individual’s risk of having or developing Type 2 diabetes in the near future. Although all participants answered these questions, the risk score was only reported for participants without previously diagnosed diabetes. The CANRISK scores were categorized into low risk (0 to 21 points), medium risk (21 to 32 points) and high risk (more than 33 points).

#### Health behaviours

The HABiT consists of questions about self-reported physical activity, diet, smoking and alcohol intake. Participants were asked how often they engage in physical activity and in sedentary behaviours (reporting the number of hours spent on the computer, TV and reading). For diet, participants were asked about their fruits, vegetables, starchy food, fatty food, sugary food and salt consumption.

#### Health literacy

Based on the  NVS-UK test, participants were shown a nutrition label and were asked to answer six questions based on the information provided. The health literacy score was determined based on the number of correct answers. Health literacy was categorized as adequate (4 to 6 points) or below adequate (0 to 3 points).

### Data analysis

Baseline characteristics of both populations were analyzed using descriptive statistics from data collected from the HABiT questionnaire. Demographics and cardiometabolic risk factors between the two provinces were compared using bivariate analysis: Chi-Square tests for categorical variables, independent sample t-tests for the continuous variables, and Mann–Whitney U tests for ordinal variables. Participants with missing responses were excluded only from the analyses of those specific items. Statistical software, IBM SPSS Version 20.0, was used to conduct the data analysis.

## Results

### Demographic characteristics

A total of 80 participant responses were obtained from Quebec for this study. This was then compared to the previously collected sample of 599 responses from Ontario. Demographic characteristics for social housing residents in Ontario and Quebec are listed in Table 1. Statistically significant differences (p < 0.05) were found in gender, education and access to a family doctor. The Ontario sample had significantly (p < 0.05) more females, a higher proportion of participants with post-secondary education (p < 0.001), and a higher proportion with access to a family doctor (p < 0.001), compared to the social housing residents in Quebec.

**Table 1:** Demographic characteristics of all study participants

Variable	Ontario (%) N = 599	Quebec (%) N = 80	P-value
*Age*	n = 599	n = 76	
< 65 years	25.4	17.1	0.271
65–74 years	28.9	26.8	
75–84 years	26.4	34.2	
> 84 years	9.3	11.8	
Gender**	n = 599	n = 80	
Male	11.3	24.4	**0.009**
Female	88.8	75.6	
*Mother’s ethnicity*	n = 588	n = 77	
White	92.0	97.4	0.506
Black	2.7	0.0	
Native	2.2	1.3	
East Asian	1.4	0.0	
South Asian	0.9	0.0	
Other non-white	0.9	1.3	
*Father’s ethnicity*	n = 588	n = 77	
White	91.8	96.1	0.749
Black	1.7	1.3	
Native	2.9	1.3	
East Asian	1.5	0.0	
South Asian	0.9	0.0	
Other non-white	1.2	1.3	
*Education*	n = 593	n = 80	
Some high school or less	45.7	68.8	** < 0.001**
High school diploma	22.8	18.8	
Some college or university	14.8	8.8	
University of college degree	16.7	3.8	
*Current marital status*	n = 592	n = 80	
Married	7.9	3.8	0.073
Living common-law	1.5	1.3	
Widower	30.2	22.5	
Separated	8.4	3.8	
Divorced	37.2	53.8	
Single, never married	14.7	15.0	
*Has family doctor*	n = 599	n = 80	
Yes	93.5	90.0	**<0.001**
*Self-reported health status*	n = 594	n = 80	
Poor	11.4	13.8	0.968
Fair	29.0	25.0	
Good	33.5	36.3	
Very good	20.7	18.8	
Excellent	5.4	6.3	
*Health literacy*	n = 232*	n = 80	
Mean score (SD)	1.57 (1.86)	1.25 (1.45)	0.119
Below adequate health literacy (scored 0 to 3)	82.8	90.0	0.122
Adequate health literacy (scored 4 to 6)	17.2	10.0	

Bolding indicates statistically significant differences between Ontario and Quebec at a threshold of p < 0.05; SD = standard deviation; *health literacy was only measured in one of the Ontario communities

^**^Gender was taken from the CANRISK Question, “Are you male or female?”

### Health-related quality of life

There was a significant difference in self-care between the provinces (p = 0.017) with Quebec social housing residents experiencing less problems with self-care as seen in Table 2. Mobility was trending towards Ontario residents having more issues (p = 0.052). Usual activities (p = 0.161), pain/discomfort (p = 0.246) and anxiety/depression (p = 0.464) showed no statistically significant differences. Visual analog scale for health state was also similar with a mean of 67.36 (standard deviation [SD] = 21.28) in Ontario and 69.23 (SD = 19.90) in Quebec.

**Table 2:** EQ5D-3L responses for all participants

Variables	Ontario (%)	Quebec (%)	P-value
	N = 599	N = 80	
*Mobility*	n = 594	n = 79	
I am confined to a bed	2.5	0.0	0.052
I have some problems walking about	57.2	49.4	
I have no problems in walking about	40.2	50.6	
*Self-care*	n = 592	n = 80	
I am unable to wash or dress myself	3.7	0.0	**0.017**
I have some problems with washing or dressing myself	15.7	8.8	
I have no problems with self-care	80.6	91.3	
*Usual activities*	n = 593	n = 77	
I am unable to perform usual activities	6.2	2.6	0.161
I have some problems with performing my usual activities	36.3	32.5	
I have no problems with performing my usual activities	57.5	64.9	
Pain/discomfort	n = 594	n = 80	
I have extreme pain or discomfort	16.3	13.8	0.246
I have moderate pain or discomfort	53.9	50.0	
I have no pain or discomfort	29.8	36.3	
*Anxiety/depression*	n = 593	n = 80	
I am extremely anxious or depressed	9.1	7.5	0.464
I am moderately anxious or depressed	36.3	33.8	
I am not anxious or depressed	54.6	58.8	
*Visual analog scale (1 to 100) of health state*	n = 590	n = 80	
Mean (SD)	67.36 (21.28)	69.23 (19.90)	0.459

Bolding indicates statistically significant differences between Ontario and Quebec at a threshold of p < 0.05; SD = standard deviation

### Knowledge of risk factors

As seen in Table 3, overall knowledge of risk factors was similar between Ontario and Quebec with no significant differences found in 14 out of 19 statements. Differences were observed for five statements: high blood pressure is a risk factor for heart attacks and strokes (p = 0.002), high blood pressure can cause other serious health problems (p = 0.012), diabetes can cause other serious health problems (p < 0.001), stressful lifestyle can contribute to high blood pressure (p = 0.016) and eating too much sugar or sweet foods is a cause of diabetes (p = 0.001).

**Table 3:** Percentage of participants that had correct responses for knowledge of risk factors

True/False Knowledge Statements	Ontario (%) N = 599	Quebec (%) N = 80	P-value
High blood pressure is a risk factor for heart attacks and strokes	93.1	82.9	**0.002**
Diabetes is a risk factor for heart attacks and strokes	75.1	73.1	0.701
High blood pressure can cause other serious health problems	91.1	82.1	**0.012**
Diabetes can cause other serious health problems	73.1	98.7	** < 0.001**
High blood pressure becomes more common as people get older	75.0	78.9	0.451
Diabetes becomes more common as people get older	69.7	64.9	0.391
You can tell you have high blood pressure because you will feel unwell	49.4	53.2	0.531
Lifestyle changes such as stopping smoking and weight loss can decrease blood pressure	82.6	86.1	0.441
Blood pressure of 140/90 is considered high	55.5	63.3	0.190
In general, the following can contribute to high BP: stressful lifestyle	92.6	84.6	**0.016**
In general, the following can contribute to high BP: too much alcohol	83.4	79.5	0.382
In general, the following can contribute to high BP: too much salt	89.1	88.6	0.889
You are at risk of developing diabetes if you are obese	86.2	80.8	0.195
Eating too much sugar or sweet food is a cause of diabetes	33.2	15.4	**0.001**
People who have family members with diabetes have an increased risk of developing diabetes	78.8	80.5	0.722
Diabetes can be cured	55.5	59.0	0.563
High blood pressure can be treated by exercise and weight loss	88.1	80.5	0.061
To reduce the risk of diabetes you need to eat well and exercise regularly	94.0	94.9	0.735
The recommended blood pressure for most adults is less than 120/80	60.2	70.9	0.067

Missing responses were coded as the respondent not giving the correct response. Bolding indicates statistically significant differences between Ontario and Quebec at a threshold of p < 0.05; SD = standard deviation

### Self-reported chronic disease and CANRISK results

Differences in self-reported chronic disease were found for blood pressure, blood sugar and cholesterol. Table 4 shows that more social housing residents in Ontario had their blood pressure checked by a health professional in the last 6 months (87.0%, p = 0.009). However, a higher percentage of Quebec social housing residents were observed to have had their cholesterol (p < 0.001) checked by a health professional in the last two years. There was no statistically significant difference observed between the CANRISK scores (p = 0.091) and interpretations (p = 0.199).

**Table 4:** Self-reported chronic diseases and CANRISK results

Variables	Ontario (%)	Quebec (%)	P-value
	N = 599	N = 80	
*Had BP taken by a health professional*	n = 596	n = 80	
Yes	98.8	98.8	0.953
*Last time BP was taken by a health professional*	n=591	n=80	
5 or more years ago	1.0	1.3	**0.009**
2 years to less than 5 years	1.5	2.5	
1 year to less than 2 years	2.2	7.5	
6 months to less than a year	8.3	12.5	
Less than 6 months	87.0	76.3	
*Told by a health professional that they had high BP or take high BP pills*	n = 599	n = 80	
Yes	63.0	58.8	0.460
*Had cholesterol checked in the past 2 years*	n = 597	n = 79	
Yes	77.4	89.2	**0.019**
*Had blood sugar levels checked in the past 2 years*	n = 599	n = 80	
Yes	76.0	81.8	0.253
*BP systolic*^a^	n = 249	n = 23	
Mean (SD)	131.32 (19.44)	131.00 (15.15)	0.939
140 and above, %	30.1	34.8	0.642
*BP diastolic*^b^	n = 229	n = 18	
Mean (SD)	76.29 (11.78)	79.33 (10.16)	0.288
90 and above, %	17.5	27.8	0.275
*Had high blood sugar levels from blood test, illness or pregnancy*	n = 580	n = 80	
Yes	30.3	32.4	0.723
*Have heart problems*	n = 599	n = 80	
Yes	28.5	27.5	0.845
*Have hypertension*	n = 599	n = 80	
Yes	54.6	52.5	0.724
*Have high cholesterol*	n = 599	n = 80	
Yes	37.9	31.3	0.248
*Have had a stroke*	n = 599	n = 80	
Yes	12.9	11.3	0.685
*Have diabetes*	n = 599	n = 80	
Yes	27.5	27.5	0.993
*CANRISK (diabetes risk)*^c^	n = 267	n = 43	
Score, mean (SD)	34.31 (8.58)	36.77 (10.26)	0.091
Risk category			
Low	3.7	0.0	0.082
Medium	41.9	32.6	
High	54.3	67.4	

Bolding indicates statistically significant differences between Ontario and Quebec at a threshold of p < 0.05; SD = standard deviation, BP = Blood Pressure; ^a^Restricted to those who knew their most recent systolic BP measurement; ^b^Restricted to those who knew their most recent diastolic BP measurement; ^c^Restricted to those who self-reported as not having diabetes already

### Modifiable risk factors

Table 5 shows that compared to social housing residents in Ontario, our study found that social housing residents in Quebec smoked more cigarettes per day (p = 0.009). In comparison, residents in the Ontario sample participated in significantly more mild and moderate physical activities per week (p = 0.007 and 0.009, respectively), however, they also spent more time on the computer (p = 0.006). There was a significant difference in frequency of fruit and vegetable consumption between provinces, with a greater percentage of Ontario social housing residents consuming fruits and vegetables every day (p = 0.006).

**Table 5:** Modifiable risk factors responses

Variables	Ontario (%)	Quebec (%)	P-value
	N = 599	N = 80	
*Physical activity for 30 min a day*	n = 591	n = 80	
Yes	46.3	36.3	0.091
*Number of times of physical activity a week*	n = 329	n = 78	
Mild, mean (SD)	5.65 (6.70)	3.49 (4.37)	**0.007**
Moderate, mean (SD)	2.46 (3.40)	1.42 (3.01)	**0.009**
Strenuous, mean (SD)	0.26 (1.00)	0.42 (1.91)	0.456
*Time spent on a computer*	n = 519	n = 39	
> 12 h	2.1	0.0	**0.006**
10 to 12 h	1.7	2.6	
7 to 9 h	3.5	10.3	
3 to 6 h	16.0	30.8	
0 to 3 h	76.7	56.4	
*Time spent in front of television*	n = 585	n = 80	
>12 h	7.9	6.3	0.136
10 to 12 h	5.5	3.8	
7 to 9 h	13.2	17.5	
3 to 6 h	41.2	53.8	
Less than 3 h	32.3	18.8	
*Time spent reading*	n = 596	n = 80	
>12 h	3.9	0.0	0.530
10 to 12 h	2.2	3.8	
7 to 9 h	4.7	8.8	
3 to 6 h	22.8	25.0	
< 3 h	66.4	62.5	
*Fruit and vegetable consumption*	n = 594	n = 80	
< 1 time /week	4.0	6.3	**0.006**
1 time /week	3.7	2.5	
2 to 3 times /week	17.2	25.0	
4 to 5 times /week	8.6	18.8	
Every day	66.5	46.3	
*Pieces of fruits and veggies a day*	n = 598	n = 80	
No pieces	7.0	6.3	0.112
1 to 2 pieces	46.3	58.8	
3 to 4 pieces	33.4	23.8	
5 to 7 pieces	10.4	11.3	
>7 pieces	2.8	0.0	
*Monitor diet to maintain healthy weight*	n = 597	n = 80	
Always	35.0	25.0	0.109
Frequently	10.7	6.3	
Sometimes	16.2	26.3	
Rarely	16.9	13.8	
Not at all	21.1	28.7	
*Starch or carbohydrate consumption*	n = 599	n = 80	
Part of every meal	15.0	12.5	0.897
Part of 1 to 3 meals /day	40.1	35.0	
1 to 3 times /week	10.7	28.7	
4 to 6 times /week	26.5	22.5	
Never	7.7	1.3	
*Fatty food consumption*	n = 598	n = 80	
Never	21.9	26.3	0.344
2 to 3 times/ month	28.6	23.8	
1 to 2 times /week	29.9	37.5	
3 to 4 times /week	10.4	11.3	
Every day	9.2	1.3	
*Sugary food consumption*	n = 597	n = 79	
Never	16.8	7.6	0.918
2 to 3 times/ month	20.9	25.3	
1 to 2 times /week	22.8	30.4	
3 to 4 times /week	15.1	25.3	
Every day	24.5	11.4	
*How often do you add salt to your food*	n = 597	n = 80	
Never	33.5	27.5	0.969
Rarely	21.6	26.3	
Sometimes	17.1	20.0	
Often	10.2	20.0	
Always	17.6	6.3	
*Type of smoker*	n = 586	n = 80	
Daily smoker	29.2	17.5	0.093
Occasional smoker	2.0	1.3	
Former smoker who quit	36.2	45.0	
Never smoked	32.6	36.3	
*Number of cigarettes per day*	n = 354	n = 50	
Mean (SD)	17.84 (13.41)	23.20 (13.54)	**0.009**
*Number of cigars per day*	n = 45	n = 50	
Mean (SD)	0.69 (2.69)	0.40 (1.98)	0.550
*Number of pipes per day*	n = 42	n = 50	
Mean (SD)	0.10 (0.48)	0.00 (0.00)	0.167
*Average number of alcoholic drinks per week*	n = 593	n = 80	
Non-drinker	75.7	82.5	0.163
1 to 5 drinks	15.2	12.5	
6 to 10 drinks	5.4	2.5	
11 to 15 drinks	2.2	1.3	
More than 15 drinks	1.5	1.3	
*5 or more drinks on one occasion in the last 12 months*	n = 591	n = 80	
Never or less than once a month	90.2	88.8	0.666
Once a month	3.4	3.8	
2 to 3 times a month	2.9	2.5	
Once a week	1.7	1.3	
More than once a week	1.9	3.8	

Bolding indicates statistically significant differences between Ontario and Quebec

## Discussion

This study explores the health profiles, health behaviours, and risk factor knowledge of social housing residents in Quebec, and compared it to previously collected data from Ontario. Literature suggested that Ontario residents have better access to family doctors [20]. However, our study found that among social housing residents, the proportion with family doctors was similar in both provinces (93% in Ontario versus 90% in Quebec). We found significant interprovincial differences in HRQoL, knowledge of risk factors, chronic disease history and monitoring, and modifiable risk factors. Furthermore, upon comparison with the 2018 CCHS, a cross-sectional survey collecting self-reported health-related information across each province [21], similarities and differences were found.

HRQoL in Ontario and Quebec was shown to be similar in the domains of problems with anxiety and depression, pain and discomfort, and usual activities. In the remaining domains, our data showed that Ontario respondents had significantly more problems with self-care than Quebec, and problems with mobility demonstrated trends in the same direction. This aligns with the Canadian Survey on Disability from 2012 which reports that the prevalence of a mobility-type disability is higher in Ontario than Quebec (66.9% vs 59.6%) [21, 22]. Though only one out of five domains showed statistical significance, Ontario was worse overall for all HRQoL domains, which highlights the different needs these populations may have. Another consideration is the provision of care that differs between provinces with respect to long-term care (LTC). The only option for those with a loss of autonomy in Ontario are publicly funded LTC facilities, which have a waitlist of 35,000 people [23]. However, in Quebec, the waitlist is closer to 3,000 as there are other models of care for those experiencing a loss of autonomy, such as smaller LTC facilities [24]. With the growing older adult population facing long waitlists for LTC and low HRQoL, interventions to support aging Ontarians to stay safe at home are necessary.

Knowledge of risk factors for chronic diseases varied between provinces. Ontario respondents knew more about the cause and effect of high blood pressure, and Quebec respondents knew more about the cause and effect of diabetes. In addition, more Ontario respondents reported getting their blood pressure checked and more Quebec respondents reported getting their cholesterol levels checked. This observed difference in knowledge and screening behaviour may be due to provincial differences in public health messaging, however future research is needed to examine this potential explanation. Although it is not the only factor responsible for an individual’s health behaviours, knowledge of chronic diseases is important for proper self-management [25]. These findings also highlight the differing knowledge gaps in social housing populations in Ontario and Quebec, which can inform topics for future health interventions or community health program adaptations.

Self-reported diabetes presented no differences between the provinces, and this aligns with CCHS findings (18.6% in Ontario, 18.5% in Quebec) [8]. We found that self-reported hypertension was not significantly different among older adults between provinces. However, the CCHS reports slightly higher rates of hypertension in Ontario (44.1% in Ontario vs 40.9% in Quebec) [8]. Of note, the rate of hypertension in our sample of social housing residents was higher than the CCHS findings, with over 50% reporting having hypertension. This demonstrates a similar need in both provincial contexts for community health interventions aimed at reducing the rates of hypertension, as it is evident that social housing populations face higher rates overall than the general population [19]. Therefore, this component of our existing Ontario health intervention would not require adaptation for the Quebec context.

Other modifiable risk factors showed significant differences between the two provinces. Ontario respondents spent more time at the computer each day and added more salt to their food. Conversely, they reported exercising more frequently, and smoking less than Quebec respondents. The 2018 CCHS similarly reports better exercise and smoking behaviours in Ontario, however it does not report on monitoring diet, adding salt or computer usage [8]. The 2004 CCHS cycle included salt intake and reported that Quebec had higher values, which is the opposite of the current study findings [26]. In the 2017 International Health Policy Survey of Older Adults, more Ontario respondents reported having a discussion with a health professional in the past 2 years around healthy eating (46.7% vs 43.1%) and physical activity (54.9% and 47.5%) [27]. Therefore, better exercise and diet monitoring behaviours in Ontario may be due to the discussions they are having about these risk factors with their family doctor or other health professionals. Further research is needed to explore these differences in modifiable risk factors between Ontario and Quebec, which would result in specific health education programming adaptations for exercise and diet in each context.

## Limitations

This study used self-reported data, which is subject to recall and social desirability biases. Consecutive sampling had to be used for better response rates in a hard-to-reach population [12]; therefore, there may be a selection bias. Also, as only a small sample of the population was surveyed, this may not be fully representative of the older adult social housing population. However, this approach was needed to collect the study data from a population that traditionally is underrepresented in research and face barriers to participation [12].

## Conclusion

Health profiles and health behaviours among older adults living in social housing in Ontario and Quebec are similar for many of the variables assessed. Differences were found in ability to conduct self care and in their rates of blood pressure and cholesterol screening. Health interventions can be used with both populations with minor adaptations. Future research on health-related community programming that targets social housing populations to address the quality of life, knowledge and modifiable risk factors would be beneficial to improve the health outcomes and well being of this population in Ontario and Quebec.

## Data Availability

The datasets generated during the current study are not publicly available to ensure confidentiality of participants but are available from the corresponding author on reasonable request.

## Abbreviations

HABiT: Health awareness and behaviour tool
CCHS: Canadian Community Health Survey
CANRISK: Canadian Diabetes Risk Questionnaire
CHEP: Canadian Hypertension Education Program
EQ5D-3L: EuroQual Five Dimensions, 3-level
NVS-UK: Newest Vital Sign UK
HRQoL: Health-related quality of life
LTC: Long-term care
